# PTRF/Cavin-1 decreases prostate cancer angiogenesis and lymphangiogenesis

**DOI:** 10.18632/oncotarget.1300

**Published:** 2013-10-04

**Authors:** Zeyad D. Nassar, Hyeongsun Moon, Tam Duong, LiQi Neo, Michelle M. Hill, Mathias Francois, Robert G. Parton, Marie-Odile Parat

**Affiliations:** ^1^ The University of Queensland, School of Pharmacy, QLD, Australia; ^2^ The University of Queensland Diamantina Institute, Translational Research Institute, QLD, Australia; ^3^ The University of Queensland, Institute for Molecular Bioscience, QLD, Australia

**Keywords:** PTRF, Caveolae, Angiogenesis, Lymphangiogenesis, Prostate Cancer

## Abstract

Caveolae are specialized plasma membrane subdomains implicated in cellular functions such as migration, signalling and trafficking. Caveolin-1 and polymerase I and transcript release factor (PTRF)/cavin-1 are essential for caveola formation. Caveolin-1 is overexpressed and secreted in prostate tumors and promotes aggressiveness and angiogenesis. In contrast, a lack of PTRF expression is reported in prostate cancer, and ectopic PTRF expression in prostate cancer cells inhibits tumor growth and metastasis. We experimentally manipulated PTRF expression in three prostate cancer cell lines, namely the caveolin-1 positive cells PC3 and DU145 and the caveolin-1-negative LNCaP cells, to evaluate angiogenesis- and lymphangiogenesis-regulating functions of PTRF. We show that the conditioned medium of PTRF-expressing prostate cancer cells decreases ECs proliferation, migration and differentiation *in vitro* and *ex vivo*. This can occur independently from caveolin-1 expression and secretion or caveola formation, since the anti-angiogenic effects of PTRF were detected in caveolin-1-negative LNCaP cells. Additionally, PTRF expression in PC3 cells significantly decreased blood and lymphatic vessel densities in orthotopic tumors in mice. Our results suggest that the absence of PTRF in prostate cancer cells contributes significantly to tumour progression and metastasis by promoting the angiogenesis and lymphangiogenesis potential of the cancer cells, and this could be exploited for therapy.

## INTRODUCTION

Experimental and clinical data indicate that the progression of prostate cancer depends on angiogenesis and lymphangiogenesis. Prostate cancer cells express angiogenesis inducers vascular endothelial growth factor (VEGF), and interleukin-8 (IL-8) [1]. Endothelial cell (EC) proliferation is stimulated by co-culture with prostate cancer cells or exposure to their conditioned medium [2]. VEGF expression in clinical samples correlates with prostate-specific antigen (PSA) levels, Gleason score and reduced survival rate [3]. Additionally, microvessel density in prostate tumors is associated positively with Gleason score and predicts survival [4;5]. Lymph nodes are the first metastasis destination for prostate cancer cells, and poor prognosis as well as shorter disease free survival are reported in patients with lymph node metastasis [6-8]. Lymphangiogenesis promotes prostate cancer metastasis to lymph nodes. Studies report an association between expression of lymphangiogenesis activators such as VEGF-C, VEGF-D and VEGFR-3 and lymph node metastasis [9-11]. Accordingly, a positive correlation was reported between peri-tumoral lymphatic vessel density and lymph node metastasis [12].

The caveola-forming protein Cav-1 is overexpressed and secreted in prostate cancer and promotes growth, metastasis, angiogenesis, and conversion to hormone-independent status (reviewed in [13]). Caveolae are 50–100 nm flask-shaped specialized plasma membrane invaginations implicated in cellular signalling, endocytosis, lipid and cholesterol homeostasis, mechanosensing, cell migration adhesion and invasion [14;15]. In addition to membrane-inserted caveolin-1 (Cav-1), recent studies showed that caveola formation and functions require cytoplasmic proteins of the cavin family, which includes four members, named cavin-1 to -4. Cavin-1, also termed polymerase I and transcript release factor (PTRF) [16;17] and in specific tissues, cavin-2 [18] are essential for caveola formation. Cav-1 recruits PTRF (as a complex with other cavins) to the plasma membrane and both Cav-1 and PTRF are present in caveolae in close proximity [17]. The absence of PTRF leads to the loss of caveolae *in vitro* and *in vivo* [16;17;19].

Two recent studies investigated the expression of PTRF in cancer and normal human prostate epithelia. One reported that PTRF was expressed in normal prostate epithelium [20] while the other, employing a larger cohort, found no PTRF in normal epithelia [21]. Importantly, both studies reported that PTRF is not expressed in prostate cancer epithelium [21;22]. Therefore, in prostate cancer, caveolin-1 is overexpressed without PTRF. This unusual imbalance between Cav-1 and PTRF expression is exemplified in the prostate cancer cell line PC3. Ectopic expression of PTRF in endogenously Cav-1-expressing PC3 restores caveola formation [17], alters the cell proteome and secretome [23], significantly reduces cell migration and protease production [24] and reduces *in vivo* tumor growth and metastasis [21]. In agreement with a protective role for PTRF in prostate cancer, PTRF down regulation in DU145 cells enhances their 3-D migration [25]. Intriguingly, co-culture with or conditioned medium from the PTRF-expressing cells DU145 are unable to stimulate lymphatic endothelial cell migration and tube formation compared with the PTRF-devoid PC3 and LNCaP, suggesting that paracrine factors promoting lymphangiogenesis may be regulated by PTRF [26].

In the present study, we tested the effect of PTRF expression in 3 prostate cancer cell lines on their angiogenesis- and lymphangiogenesis-promoting phenotype using *in vitro*, *ex vivo* and *in vivo* assays.

## RESULTS

### Effect of PTRF expression on Cav-1 expression and secretion in PCa cells

**Figure 1:**
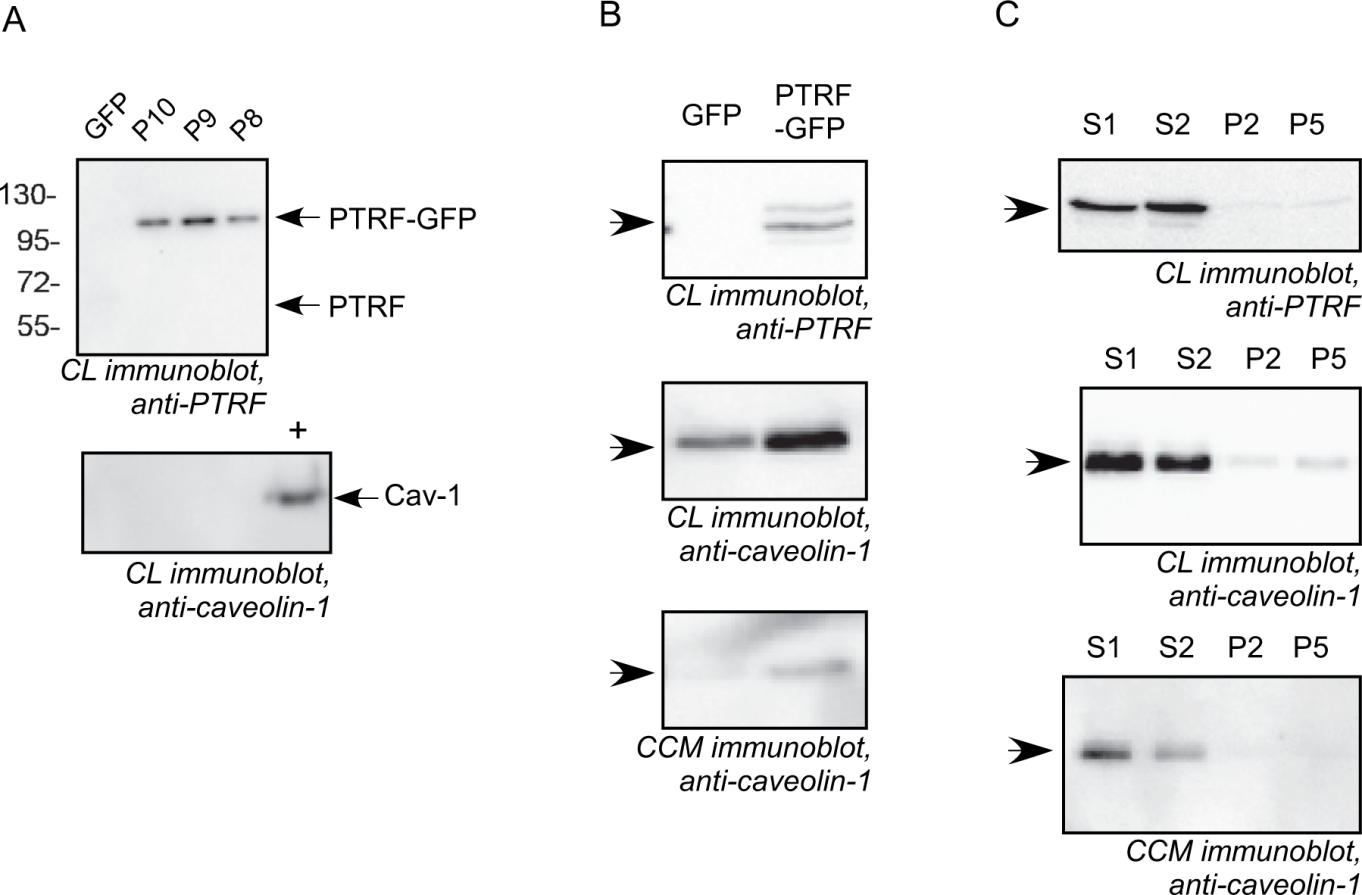
Cav-1 and PTRF expression in prostate cancer cell lines Cell lysates (CL) from LNCaP, PC3 and DU145 and concentrated conditioned medium (CCM) from DU145 and PC3 cells were separated by SDS-PAGE and subjected to immunoblot analysis using anti-PTRF or anti-caveolin-1 antibodies. (A) LNCaP cells clones stably expressing GFP or PTRF-GFP (P8, P9 and P10). A Cav-1 positive control cell lysate (+) was used for the Cav-1 blot. (B) PC3 pooled cells stably expressing PTRF-GFP or GFP. (C) DU145 cell clones stably transfected with scrambled shRNA (S1 and S2 clones) or with PTRF shRNA (P2 and P5 clones).

We manipulated the expression of PTRF in three prostate cancer cell lines, namely PC3 cells (which express abundant Cav-1 but no PTRF), LNCaP (which produce neither Cav-1 nor PTRF) [24] and DU145 (which express both Cav-1 and PTRF) [17]. The ectopic expression of PTRF in LNCaP cells and PC3, and PTRF down regulation in DU145 cells were confirmed using Western blotting of the cell lysates (figure 1). The expression of PTRF in LNCaP cells did not lead to the expression of endogenous Cav-1. However, the expression of PTRF in PC3 cells increased the amount of Cav-1 in both cell lysate and conditioned medium. Down regulation of PTRF in DU145 resulted in reduction of Cav-1 in the cell lysate as well as in the conditioned medium.

**Figure 2:**
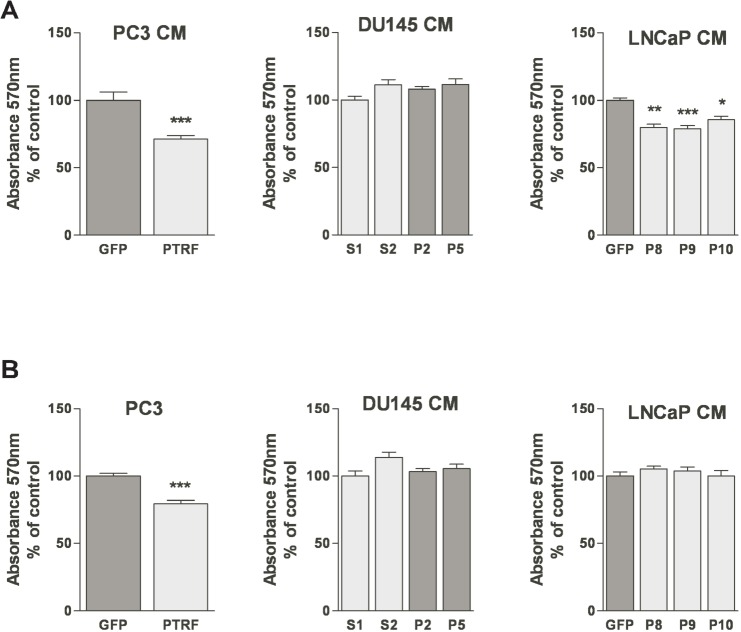
Effect of PTRF expression in prostate cancer cells on endothelial cell viability (A) BAEC and (B) LEC proliferation after treatment with various prostate cancer cell-conditioned medium was tested using the MTT assay. Results are reported as percent of the viability of ECs to the control cells (*n*=3) *P<0.05, **P<0.01 and ***P<0.001.

### Effect of PTRF expression in PCa on EC and LEC proliferation

ECs are normally quiescent and divide rarely with an average turnover rate of once every three years [27]. Yet, upon angiogenic induction, the proliferation rate of ECs increases substantially [27]. The effect of PTRF expression by prostate cancer cells on their ability to elicit EC and LEC proliferation was evaluated using the MTT assay after 48h of exposure to prostate cancer cell conditioned media. While PTRF down-regulation in DU145 cells did not significantly change BAEC viability, the conditioned medium of PTRF- expressing LNCaP and PC3 cells reduced BAEC viability significantly compared to conditioned media of the control cells devoid of PTRF. There was no significant difference between LEC proliferation in conditioned media from either LNCaP or DU145 cells, but the medium of PTRF-expressing PC3 cells reduced LEC viability significantly compared to that of control PC3 cells.

### PTRF expression level in prostate cancer cells modulates their production of endothelial and lymphatic chemotactic factors

**Figure 3:**
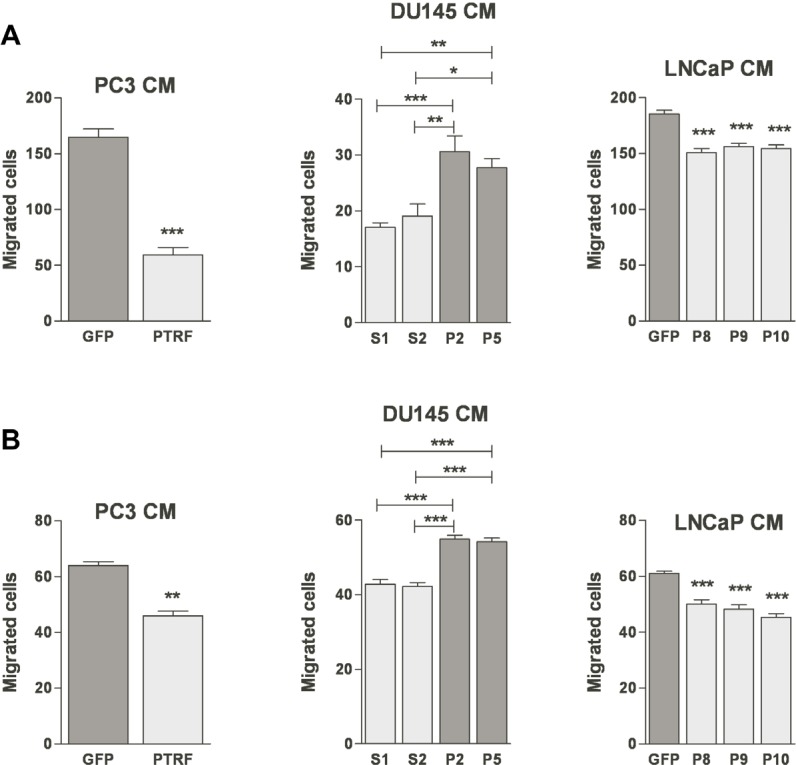
Effect of PTRF expression in prostate cancer cells on endothelial cell three-dimensional chemotaxis (A) BAEC and (B) LEC were tested for their ability to migrate in a modified Boyden chamber assay toward various prostate cancer cell-conditioned media. Results are reported as percent of the migration to the control cells (*n*=3-5) *P<0.05, **P<0.01 and ***P<0.001.

EC migration toward a growth factor concentration gradient is a crucial step in tumour angiogenesis and lymphangiogenesis. Ectopic expression of PTRF in prostate cancer cells decreased BAEC and LEC chemotaxis significantly. Their migration in the Boyden chamber assay toward the conditioned medium of PTRF-expressing LNCaP or PC3 was significantly lower than toward the conditioned medium of control cells. Accordingly, down-regulation of PTRF expression in DU145 cells enhanced BAEC and LEC transmigration towards DU145 conditioned medium significantly (figure 3).

### Effect of PTRF expression in prostate cancer cells on blood and lymphatic endothelial cell random migration

**Figure 4:**
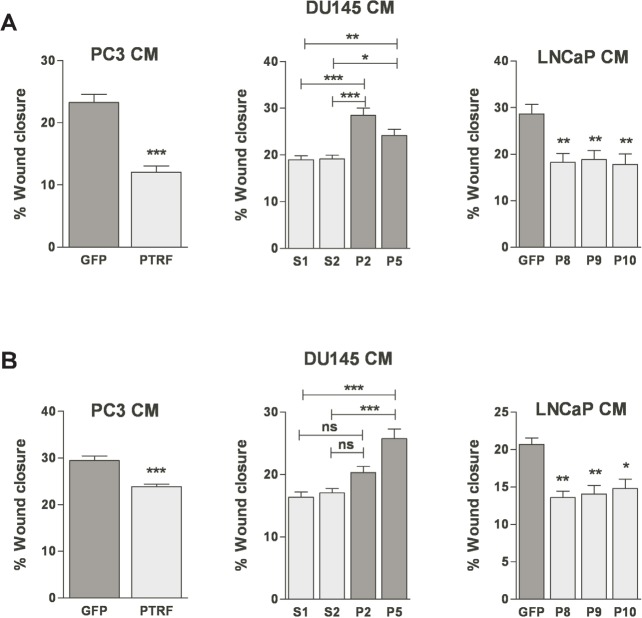
Effect of PTRF expression in prostate cancer cells on endothelial cell two-dimensional chemokinesis (A) BAEC and (B) LEC were tested for their ability to migrate randomly in the wound healing assay. Wounded EC monolayers were exposed to various prostate cancer cell-conditioned media for 6 h. Results are reported as percent of wound closure (*n*=3-6) *P<0.05, **P<0.01 and ***P<0.001.

In order to confirm the effect of manipulating PTRF expression in prostate cancer cells on EC migration, we tested the conditioned media of prostate cancer cells expressing or devoid of PTRF on EC in the scratch wound assay. Ectopic expression of PTRF in LNCaP and PC3 cells reduced the wound closure of both BAECs and LECs when compared with control PC3 and LNCaP cells. Likewise, BAEC and LEC exposed to the conditioned medium of PTRF–down regulated DU145 cells migrated faster than those exposed to conditioned medium of PTRF-expressing cells (figure 4).

### PTRF expression in prostate cancer cells modulates EC and LEC tube formation

**Figure 5:**
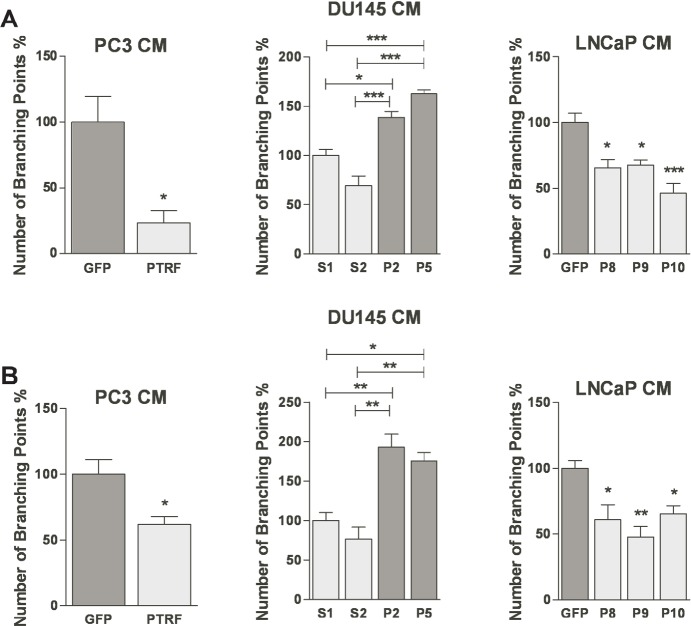
Effect of PTRF expression in prostate cancer cells on in *vitro* angiogenesis and lymphangiogenesis (A) BAEC and (B) LEC were tested for their ability to differentiate into tube-like structures on Matrigel^™^ when exposed to various prostate cancer cell-conditioned media for 6 hr. Results are reported as percent of branching points compared to control cells (*n*=3) *P<0.05, **P<0.01 and ***P<0.001.

One of the established characteristics of ECs is their ability to form capillary-like structures *in vitro* rapidly when plated on top of basement membrane extracellular matrix. Upon plating on extracellar matrix components, ECs elaborate dynamic cellular projections and then form tubule-like structures in a multi-step process that requires cell adhesion, migration, protease secretion and tube formation [28]. BAECs and LECs exposed to conditioned medium of PTRF-expressing PC3 or LNCaP cells produced incomplete tubules compared to those exposed to media from control cells, but ECs treated with medium of PTRF–down regulated DU145 cells exhibited a more differentiated phenotype and more branching points compared to cells exposed to medium from PTRF-expressing DU145 (figure 5).

### PTRF expression in prostate cancer cells modulates their ability to regulate *ex vivo* angiogenesis

**Figure 6:**
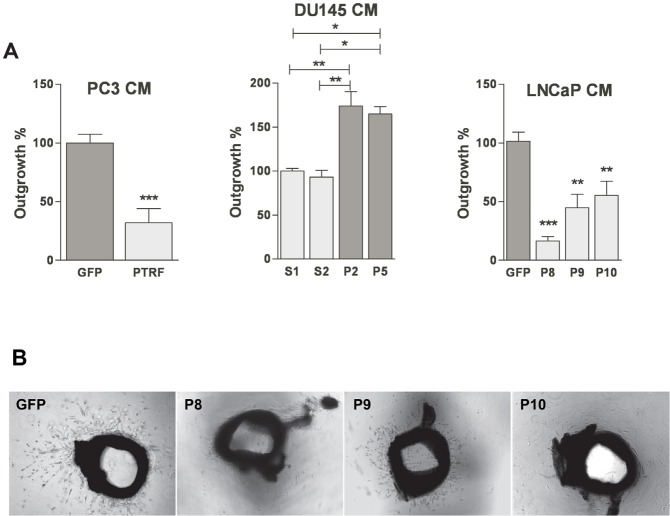
Effect of PTRF expression in prostate cancer cells on *ex vivo* angiogenesis (A) Quantification of the growth of tube-like structures from aortic ring explants exposed for 5 days to various prostate cancer cell-conditioned media supplemented with 20 ng/mL of VEGF. Results are reported as percentage of growth distance to the control (*n*=4-6) *P<0.05, **P<0.01 and ***P<0.001. (B) Representative micrographs of aortic ring explants on day 5.

We tested the effect of conditioned media from prostate cancer cells on aortic ring explants outgrowth towards VEGF. Media from PTRF-expressing PC3 or LNCaP cells induced a significant reduction of the length of sprouting tube-like structures compared to media of control cells (figure 6). Similarly, aortic rings placed in conditioned media of PTRF-down regulated DU145 cells grew longer structures than rings exposed to conditioned media of control DU145 cells.

### PTRF impairs angiogenesis and lymphangiogenesis in prostate cancer *in vivo*

**Figure 7:**
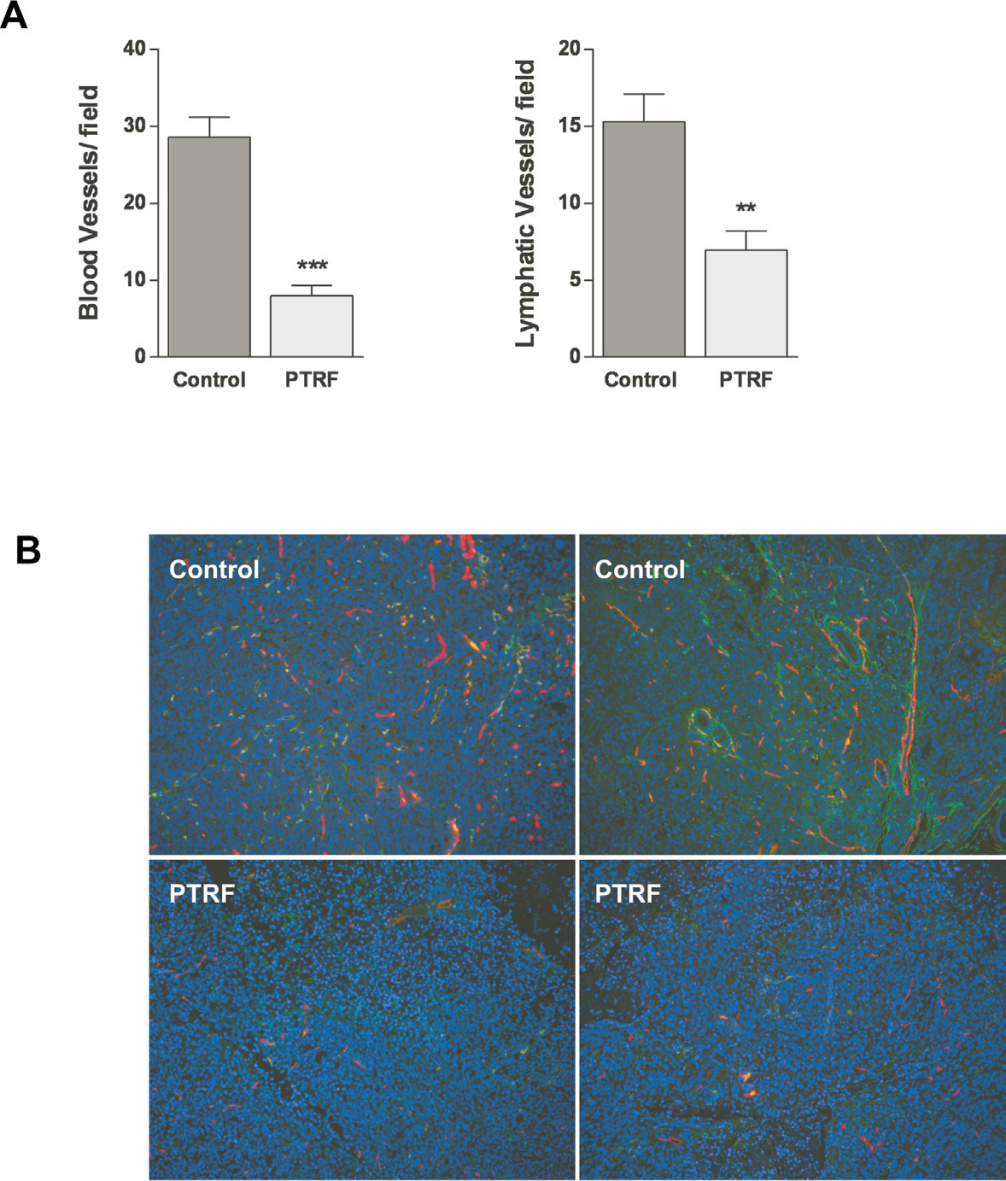
Effect of PTRF expression in PC3 prostate cancer cells on tumour angiogenesis and lymphangiogenesis *in vivo* (A) Quantitation of blood and lymphatic vessel density in tumours in five randomly selected areas, expressed as number of vessels per field (n=8) **P<0.01 and ***P<0.001. (B) Representative immunofluorescence in sections of PC3 tumors using the lymphatic specific marker podoplanin (green) and the panendothelial cell marker endomucin (red).

To confirm the antiangiogenic and antilymphangiogenic activities of PTRF *in vivo*, we used an orthotopic prostate cancer xenograft mouse model previously employed to show that PTRF expression in PC3 cells reduces *in vivo* tumor growth and metastasis [21]. Parraffin-embedded sections of 16 tumours generated from PTRF-expressing or control PC3 cells [21] were analysed by immunofluorescence using the lymphatic-specific marker podoplanin and the panendothelial cell marker endomucin (figure 7B). Quantitation of blood and lymphatic vessel density revealed that PTRF expression reduces the formation of blood and lymphatic vessels in prostate tumors by about 72% and 55%, respectively (figure 7A).

## DISCUSSION

The role of the caveola-forming proteins caveolin-1 and, most recently, cavin-1 in cancer has been evaluated in *in vitro*, preclinical and clinical studies and while multiple studies find differential expression in cancer, they differ in their conclusions, leading to the hypotheses that there may be tumour type and tumour stage differences, primary versus metastatic and cancer cell versus stromal cell specificity in expression pattern and functionality [21;29-35]. The evaluation of cancer clinical samples and cell lines has begun to unveil a potential tumour-suppressor role for PTRF, which was reported to be down-regulated in breast cancer tissues and cells [36], non-small-cell lung cancer extracts [37] and tumorigenic human bronchial epithelial cells [38]. In contrast, PTRF expression was recently documented to increase with the metastatic potential of pancreatic cancer cells [39].

We have previously demonstrated that PTRF expression in prostate cancer cells reduces their secretion of proteases, cytokines, and growth regulatory proteins [23;24], significantly reduces cell migration [24;25] and reduces *in vivo* tumor growth and metastasis [21]. Our current results show that expression of PTRF in prostate cancer cells further reduces their angiogenic and lymphangiogenic potential by inhibiting essential steps of these processes such as blood and lymphatic EC proliferation, migration, and tube formation. Conversely, down-regulation of PTRF in prostate cancer cells increases these activities. Thus, all reports so far concur that PTRF expression prevents prostate cancer aggressiveness. It is important to note that our experiments focus on prostate cancer but our results do not preclude a similar role for PTRF in other tumor types.

Prostate cancer is characterized by overexpression and secretion of the tumor-promoting protein caveolin-1 (reviewed in [13]). In most tissues, the expression of PTRF is proportional to that of caveolin-1 [17-19] either because in the absence of PTRF, caveolin-1 undergoes lysosomal degradation [17] or due to co-regulation at the transcriptional level [40]. However, despite elevated caveolin-1, prostate cancer cells in clinical samples lack PTRF expression [21;22]. In the absence of PTRF caveola cannot form, and ectopic expression of PTRF restores caveola formation in caveolin-1-rich PC3 prostate cancer cells [17]. Accordingly, in PTRF- down regulated DU145, caveolin-1 expression is drastically decreased and the number of caveolae reduced to ~4% of the control, scramble shRNA transfected cells (data not shown). It can therefore be proposed that PTRF modulates prostate cancer cell secretion of factors regulating angiogenesis or lymphangiogenesis, via caveola-dependent mechanisms, for example changes in cellular trafficking leading to altered secretome or prostasome composition [23]. However, our experiments were designed to test this hypothesis by expressing PTRF in both caveolin-1-positive PC3 cells (which then form caveolae [17]) and LNCaP cells that lack caveolin-1 (and thus do not form caveolae). In both cell lines the angiogenic and lymphangiogenic potential were similarly decreased by PTRF expression, thereby ruling out the involvement of caveolae *per se* in the observed effects.

The ability of PTRF to modulate prostate cancer angiogenesis and lymphangiogenesis results from altered paracrine factors from the prostate cancer cells that act on the blood or lymphatic endothelial cells. We have previously shown that PTRF expression reduces MMP9 production by PC3 cells independently of caveola formation [24]. We have also documented that PTRF reduces prostasome secretion [23] and IL6 production *in vitro* and *in vivo* [21;23]. These could all explain the changes in angiogenesis that we detected. Alternatively, an attractive secreted protein that could mediate the effect of PTRF on angiogenesis is caveolin-1, which is increased in the serum of prostate cancer patients [41], has been shown to exert paracrine proangiogenic effects [42], and can be antagonized by caveolin-1 antibody [43;44].

A hypothesis put forward to explain the role of PTRF in prostate cancer has been the potential for PTRF to trap caveolin-1 in caveolae and thereby reduce its secretion [23]. We assessed the consequences of modulating PTRF expression on the levels of cellular and secreted caveolin-1. In PC3, expression of PTRF increases caveolin-1 in cell lysates, presumably via stabilisation [17] but in LNCaP the expression of PTRF did not lead to endogenous caveolin-1 expression. In both DU145 and PC3 cells, we show that the absolute amount of caveolin-1 secreted increases, rather than decreases, with PTRF expression. While this does not preclude the possibility that the ratio of secreted caveolin-1 to cellular caveolin-1 is reduced when PTRF allows caveola formation, in our experiments the protection afforded by PTRF in terms of angiogenesis and lymphangiogenesis cannot be attributed to the amount of caveolin-1 that the prostate cancer cells secrete.

Taken together, the results obtained from three prostate cancer cell lines with a variety of *in vitro, ex vivo* and *in vivo* angiogenesis and lymphangiogenesis models show that PTRF expression can reduce prostate cancer new vessel formation, in a fashion that does not require caveola formation. To date, it is still unclear whether PTRF expression is down regulated in prostate epithelia when cancer progresses [22] or whether PTRF is not expressed in either normal or malignant prostate epithelia [21]. In light of the other previously demonstrated protective effects of PTRF in prostate cancer, and of its reduced expression in the stroma of prostate tumours [21], PTRF expression might provide a new way to target prostate cancer.

## MATERIALS AND METHODS

### Reagents

DMEM, DMEM/F12, RPMI 1640, penicillin/streptomycin, G418, trypsin, foetal bovine serum (FBS), glutamine and sodium pyruvate were purchased from Invitrogen (Life Technologies, Mulgrave, VIC, Australia). Non-essential amino acids (NEAA) were from Lonza (Mount Waverley, VIC, Australia). Aprotinin, leupeptin, IGEPAL, Hematoxylin, 3-(4,5-dimethylthiazol-2-yl)−2,5-diphenyltetrazolium bromide (MTT), collagen from rat tail, DAPI and dimethyl sulfoxide (DMSO) were obtained from Sigma-Aldrich (Castle Hill, NSW, Australia). Matrigel™ (Basement membrane matrix) and reduced growth factor Matrigel™ (RGF Matrigel) were from BD Biosciences (North Ryde, NSW, Australia). Human vascular endothelial growth factor (VEGF) was obtained from R&D Systems (Waterloo, NSW, Australia). Permount mounting medium and PVDF membranes were supplied by Fisher Scientific (Scoresby, VIC, Australia). Rabbit polyclonal anti-caveolin-1 was from BD Biosciences (North Ryde, NSW, Australia) and anti-PTRF from ProteinTech (Dural, NSW, Australia). Syrian hamster anti-podoplanin was from Bioclone Aust (South Yarra, VIC, Australia) and rat monoclonal anti-Endomucin from (Santa Cruz Biotechnology Inc).

### Cells and culture conditions

Human prostate adenocarcinoma cells derived from lymph node metastasis (LNCaP), brain metastasis (DU145) and bone metastasis (PC3) were transfected as follows. Pooled PC3 cells stably expressing GFP-tagged PTRF or control cells stably expressing GFP alone were previously described [24]. LNCaP clones expressing GFP-tagged PTRF (LNCaP-P8, -P9 and P10), or control cells expressing GFP alone (LNCaP-GFP) were generated by transfection using transpass D2 reagent (New England Biolabs, Arundel, QLD, Australia) and G418 selection. DU145 with reduced PTRF expression were established by stably transfecting cells with shRNA to PTRF or control shRNA as described previously [17] and selecting with G418. Two clones each of PTRF down-regulated cells (DU145-P2 and DU145-P5) and control cells (DU145-S1 and DU145-S2) have been previously described [25].

DU145 and LNCaP cells were propagated in RPMI-1640 medium supplemented with 10% (v/v) FBS, 100 i.u./ml penicillin, 100 μg/ml streptomycin and 375 μg/ml G418. PC3 cells were maintained in RPMI-1640 medium supplemented with 5% (v/v) FBS, 100 i.u./ml penicillin, 100 μg/ml streptomycin and 375 μg/ml G418. Bovine aortic endothelial cells (BAECs) were maintained in DMEM/F12 medium supplemented with 5% FBS, 100 i.u./ml penicillin and 100 μg/ml streptomycin. Lymphatic endothelial cells (LECs) isolated from mice expressing temperature-sensitive SV40 large T (*H-2K^b^-ts*A58) [45] were maintained in DMEM medium supplemented with 10% FBS, 100 i.u./ml penicillin, 100 μg/ml streptomycin, 1% non-essential amino-acids, 1% sodium pyruvate and 1% glutamine. LECs were cultured in 8% CO_2_ in a humidified atmosphere at 33°C. Other cell lines were cultured in 5% CO_2_ in a humidified atmosphere at 37°C.

### Conditioned medium preparation

Cells were grown in a 10 cm dish to 70% confluence, washed twice with PBS and then incubated with 6 ml of serum-free medium for 48 h. The cell-conditioned medium was collected, centrifuged at 400 × g for 5 minutes to remove cells and debris, and the supernatant stored at -20 °C.

### Immunoblotting

Prostate cancer cells were washed twice with PBS and lysed for 20 min at 4 °C in 50 mM Tris, pH 8, 100 mM NaCl, 0.5% sodium deoxycholate, 1% (v/v) IGEPAL, 80 nM aprotinin and 2 μM leupeptin. The lysate was centrifuged at 9,000 × g for 20 min at 4 °C. Conditioned media from PC3 cells and DU145 cells were concentrated using ultrafiltration devices with a 3 kDa MW cut-off (Amicon Ultra Centrifugal Filters, Millipore Corporation, Kilsyth, VIC, Australia). Equal amounts of protein were separated by SDS-PAGE and transferred to PVDF membranes. Non-specific binding sites were blocked using 3% (w/v) BSA or 5% (w/v) of non-fat dry milk in PBS. The target proteins were probed with the appropriate primary antibodies and donkey anti-rabbit horseradish peroxidase-conjugated secondary antibody, (GE Healthcare, Sydney, NSW, Australia) via chemiluminescence with a VersaDocTM 4000 imaging system (Bio-Rad Laboratories Inc., Gladesville, NSW, Australia).

### Cell proliferation assay

The effect of prostate cancer cell conditioned media on BAEC and LEC proliferation was evaluated using the 3-(4,5-dimethyl-2-thiazoyl)-2,5-diphenyl-2H-tetrazolium bromide (MTT) assay as described previously [46]. Briefly, BAECs or LECs were seeded at 10×10^3^ per well in 100μL of DMEM/F12 (5% serum) and DMEM (10% serum), respectively. Cells were incubated 18h to allow attachment. Cells were then washed twice with PBS and treated with prostate cancer cell-conditioned medium for 48 h. Each sample was tested in triplicate. 100 μL of MTT solution (0.5mg/mL in 5% serum medium) were added and incubated for an additional 3h. Subsequently, the medium was aspirated, and 100μL dimethyl sulfoxide (DMSO) were added. After incubation for 5 min, the absorbance at 590 nm was measured. The results are presented as percent of the viability of control cells ± SEM.

### Transmigration Assay

The 3-D transmigration assay was conducted using 48-well Boyden chambers. Briefly, polycarbonate membranes (8-μm pores) were coated with rat tail collagen type 1 (100 μg/ml in 0.2N acetic acid) and used to separate the lower chambers containing the prostate cancer cell conditioned medium from the upper chambers where the BAECs or LECs (30 × 10^3^/ml) were placed within serum-free medium. The chambers were incubated for 4 h. The cells remaining on the upper face of the membrane were scraped. The membranes were then fixed, stained with hematoxylin overnight, and mounted using permount mounting medium. The migrated cells were counted microscopically [47]. Results were reported as percent of the migration to the control cells ± SEM.

### Wound Healing Assay

BAECs or LECs were plated in 24 well plates until the formation of a confluent monolayer, after which a wound was created with a micropipette tip. The cells were then exposed to prostate cancer cell conditioned medium. The wounds were photographed promptly after wound creation, and after 6 h. The width of the cell-free wound was measured using ImageJ software (National Institutes of Health, Bethesda, MD). The results were expressed as a mean percentage of wound closure ± SEM. The percentage of wound closure was calculated according to the equation: % wound closure = ((D_0_-D_6_) / D_0_)) * 100 Where D_0_ is the wound width at 0h and D_6_ is the wound width at 6h. Using the MTT assay, we controlled that at 6h there was no influence of the conditioned media on cell viability, thereby ensuring that the wound closure was due to migration rather than proliferation.

### Tube Formation Assay

Fifty microliters of Matrigel™ matrix were transferred into each well of a 96-well plate and allowed to polymerize for 45 min at 37°C. BAECs or LECs were trypsinized and seeded in 100 μl of prostate cancer cell conditioned medium at 5,000 cell/cm^2^. After 6 h, tubular structures were imaged and the number of branching points was quantified. The number of branching points is presented as a percent of control cells ± SEM.

### *Ex vivo* mouse aortic ring assay

The mouse aortic ring assay was conducted as described previously [48]. The procedure was approved by the Animal Ethics Committee of the University of Queensland. Aortas were excised from C57BL/6 mice; the surrounding tissues were cleaned out. The aortas were sliced into approximately 1 mm-thick rings. The rings were embedded in 10 μL of LGF Matrigel™ in P35 dishes. After Matrigel polymerization, 800 μl of conditioned medium containing VEGF 20 ng/mL were added to the dishes. After 5 days, the length of blood vessels outgrowing from the primary ring explants was measured using ImageJ software (National Institutes of Health, Bethesda, MD) [49]. The growth distance of at least twenty tube-like structures per ring selected at regular intervals around the rings was measured. Growth distances were reported as the percentage of the growth distance of each cell line's respective control ± SEM.

### Immunohistochemical staining for endomucin and podoplanin

Experiments were approved by the University of Queensland Animal Ethics Committee. Orthotopic prostate tumour xenografts were generated in 7-week-old male NOD.CB17-Prkdc^scid^ mice by injecting control PC3 or PTRF-expressing PC3 (5 × 10^5^ in 20 μl PBS) [21]. After 6 weeks, prostate tumours were collected, and tissues were fixed in 10% buffered formalin. The paraffin-embedded tissues were sectioned (3 μm), deparaffinised in xylene and rehydrated with descending concentrations of ethanol. For antigen retrieval, slides were heated in Antigen Unmasking Solution-Low pH (Vector laboratories, East Brisbane, QLD, Australia). After cooling and washing in PBS, slides were incubated with blocking solution [100 mmol/L maleic acid pH 7.4, 10% horse serum in PBS containing 0.1% Triton X-100 (PBSTx)] at room temperature for 1 h. Individual tissue sections were then treated with primary antibody against podoplanin (1:500 v/v in PBSTx) and endomucin (1:200 v/v in PBSTx) at 4°C for 18h. Slides were incubated with the secondary antibodies (1:200 v/v in PBSTx) for 1 h at room temperature. Nuclei were visualized using DAPI. The quantification of the lymphatic vessel and blood vessel densities was performed as described previously [50]. In brief, 5 random fields for each slide were photographed at 10X magnification, micrographs were coded using LVAP plug-in (Image J software) and overlaid with a 1 inch^2^ grid. The number of lymphatic vessels (double stained with endomucin and podoplanin) and blood vessels (stained with endomucin) per square was counted and averaged for an entire slide (n=8).

### Statistical analysis

All values are shown as mean ± SEM. Comparisons among groups were done via student t-test or one way analysis of variance (ANOVA) with appropriate post-test. *P* < 0.05 was considered significant.
